# Timing of Single-Tooth Implant Rehabilitation and Periapical Inflammation Severity: A Retrospective Study Using the DAIS System

**DOI:** 10.3390/diagnostics15202597

**Published:** 2025-10-15

**Authors:** Pascal Grün, Marius Meier, S. M. Ragib Shahriar Islam, Lilli Rödermund, Ditjon Bytyqi, Flora Turhani, Maximilian Jung, Sebastian Fitzek, Margit Mostegel, Dritan Turhani

**Affiliations:** 1 Center for Oral and Maxillofacial Surgery, Department of Dentistry, Faculty of Medicine and Dentistry, Danube Private University, Steiner Landstraße 124, 3500 Krems an der Donau, Austria; 2 Clinical Application of Artificial Intelligence in Dentistry (CAAID) Group, Department of Dentistry, Faculty of Medicine and Dentistry, Danube Private University, Steiner Landstraße 124, 3500 Krems an der Donau, Austria; 3 Austrian Center for Medical Innovation and Technology, 2700 Wiener Neustadt, Austria; 4 Research Center for Clinical AI-Research in Omics and Medical Data Science (CAROM), Department of Medicine, Danube Private University (DPU), 3500 Krems an der Donau, Austria; 5 Center for Medical Physics and Biomedical Engineering, Medical University Vienna, 1090 Vienna, Austria; 6 Medical Image Analysis & Artificial Intelligence (MIAAI) Group, Faculty of Medicine and Dentistry, Danube Private University, Steiner Landstrasse 124, 3500 Krems an der Donau, Austria; 7 ADK Diagnostics GmbH, Panikengasse 45, 1160 Vienna, Austria

**Keywords:** single-tooth implant restoration, periapical inflammation, DAIS, treatment timing, retrospective analysis

## Abstract

**Objective**: This retrospective study investigated the relationship between the timing of single-tooth implant-supported restorations—including the interval from tooth extraction and socket preservation to implant placement and final prosthetic restoration—and the severity of periapical inflammation, as classified by the Dental Apical Inflammation Score (DAIS). **Methods**: A total of 87 patients were included (DAIS 1: 8; DAIS 2: 14; DAIS 3: 1; DAIS 4: 64). Procedural intervals (extraction, socket preservation, implant placement, and prosthetic restoration) were analyzed alongside histological assessment of periapical inflammation. Clinical parameters such as tooth location, endodontic treatment status, patient age, and sex were examined using ANOVA, chi-square tests, and Pearson’s correlation analysis. **Results**: An effective sample size of N = 86 (excluding the single DAIS 3 case) was included in the parametric analysis. No significant differences in procedural timing were found across DAIS groups for the intervals between extraction and implant placement (F(2, 83) = 0.338, *p* = 0.714) or restoration (F(2, 83) = 1.016, *p* = 0.367). Tukey’s HSD post hoc analysis showed no pairwise group differences. Histological diagnosis was not significantly associated with DAIS (χ^2^(6) = 7.00, *p* = 0.321), though small subgroup sizes warrant interpretive caution. A significant association was identified between DAIS score and tooth location (χ^2^(3) = 11.79, *p* = 0.008). Patient age showed a weak but significant positive correlation with DAIS (r = 0.222, *p* = 0.039). No significant associations were found for endodontic status (χ^2^(3) = 2.54, *p* = 0.468) or sex (χ^2^(3) = 2.63, *p* = 0.452). Histological assessment revealed that most specimens represented radicular cysts with varying proportions of acute and chronic inflammatory infiltrates, consistent with the DAIS classification. **Conclusions**: Procedural timing did not significantly differ between DAIS groups. However, the observed associations with tooth location and patient age may warrant further investigation into their potential relevance for treatment planning. These findings suggest that implant timing may not need to be substantially modified according to DAIS severity alone, but that anatomical site and patient age should be considered during clinical decision-making.

## 1. Introduction

Periapical lesions are among the most frequent sequelae of pulpal and endodontic disease and represent a major reason for tooth extraction [1,2,3]. Radicular cysts account for a substantial proportion of these lesions, often reported to comprise between 6% and 55% of periapical pathologies [4,5]. Although periapical radiolucencies are commonly assessed radiographically, multiple studies have shown that radiographs alone cannot reliably differentiate radicular cysts from granulomas or other lesions [6,7,8]. As a result, histopathological analysis remains the diagnostic gold standard for periapical pathology [9,10].

Following tooth extraction, the alveolar ridge undergoes dimensional remodeling, with significant reductions in vertical and horizontal dimensions during the first six months [11,12,13]. Such resorption may compromise implant positioning, primary stability, and esthetic outcomes [14,15]. To counteract these changes, socket preservation techniques using bone grafts and barrier membranes have been established [16,17]. A wide variety of grafting materials have been introduced, including autografts, allografts, xenografts, and alloplasts, each with specific advantages and limitations [18,19]. Among these, demineralized freeze-dried bone allografts (DFDBA) are widely applied due to their combined osteoconductive and osteoinductive properties [20,21]. Similarly, resorbable collagen membranes, such as pericardium or dermis-derived barriers, are frequently employed to stabilize graft material and support guided bone regeneration [22,23]. While these techniques are effective in maintaining ridge volume, their role in influencing the timing of subsequent implant placement in sites affected by apical pathology is less well understood.

The Dental Apical Inflammation Score (DAIS) was recently proposed as a histopathological classification system that distinguishes inflammation severity based on acute and chronic components [24]. Compared to purely radiographic or clinical systems, DAIS provides a more objective and biologically meaningful framework for grading apical inflammation [25]. Despite this, its potential influence on clinical decision-making—particularly regarding implant timing—remains unclear.

To date, no study has systematically assessed whether the severity of histologically confirmed apical inflammation correlates with the intervals between extraction, implant placement, and prosthetic restoration. Understanding this relationship is clinically relevant, since clinicians frequently face the dilemma of whether to delay implant placement in sites with high inflammatory burden [26,27,28].

We therefore aimed to investigate whether DAIS severity is associated with procedural timing of implant placement and restoration. We hypothesized that higher DAIS scores would be linked to longer procedural intervals. Additionally, we explored whether patient-related variables (age, sex, tooth location, endodontic status) influence the degree of apical inflammation.

## 2. Methods

### 2.1. Study Design

This retrospective cohort study analyzed clinical and histological data from patients who underwent dental implant procedures between 2020 and 2024. The primary objective was to investigate the relationship between apical inflammation severity, as classified by the Dental Apical Inflammation Score (DAIS; range 1–4), and the timing between key procedural stages—namely, tooth extraction, socket preservation, implant placement, and final prosthetic restoration.

The study protocol was reviewed and approved by the Committee for Scientific Integrity and Ethics at the Danube Private University (DPU) in Krems, Austria (Approval Code: GZ: DPU-EK/066, Approval Date: 30 April 2024).

The study was conducted in accordance with the Declaration of Helsinki and its subsequent amendments. Reporting followed the STROCSS 2024 criteria [29].

Between January 2020 and March 2024, a total of 87 patients were retrospectively identified through the digital patient management system at the Center for Oral and Maxillofacial Surgery, Danube Private University.

Inclusion criteria were:

- Age ≥ 18 years
- Indicated for single-tooth extraction with apical pathology
- Underwent socket preservation and subsequent implant placement
- Availability of histopathological evaluation and complete clinical records Exclusion criteria included systemic inflammatory diseases (except for descriptive analysis), immunosuppressive therapy, pregnancy, active periodontitis, and missing procedural data.

The dataset included the following parameters:

- Patient demographic data
- Endodontic status of the extracted tooth (vital vs. previously treated)
- Surgical procedure dates (extraction, socket preservation, implant placement)
- Histopathological results and corresponding DAIS score
- Dates of prosthetic restoration

All patients provided written informed consent for the use of their medical and dental records. Medical history, medications, and radiographic findings were recorded for each patient. Radiographic diagnostics included panoramic radiographs and CBCT imaging (Orthophos SL 3D; Dentsply Sirona, Charlotte, NC, USA; 60–90 kVp, 3–16 mA, FOV ø5 × 5.5 cm, dose: 3–20 μSv). Patients were positioned using a three-point fixation system with alignment to the Frankfort horizontal and midsagittal planes.

All patients underwent single-tooth extraction with socket preservation. Periapical tissue was collected and histologically assessed using the DAIS system. Various implant systems were used depending on clinical indications, anatomical constraints, and prosthetic requirements. The selection was made by the operating surgeon based on available bone volume, bone quality, patient-specific preferences (e.g., zirconia vs. titanium), and previous experience with each system.

The following implant types and manufacturers were used:

- BEGO Semados^®^ S/SC/SCX, BEGO Implant Systems GmbH & Co. KG, Bremen, Germany
- Straumann^®^ BLX/BL, Institut Straumann AG, Basel, Switzerland
- 3i T3^®^ and 3i T3^®^ PT, ZimVie Inc., Westminster, CO, USA
- Biomet 3i^®^ Certain^®^, Boss implant, ZimVie Inc., Westminster, CO, USA
- SDS Zirconia^®^, Swiss Dental Solutions AG, Kreuzlingen, Switzerland

A total of 86 implants were placed using a standard drilling protocol, irrespective of implant brand, with minimal insertion torque > 30 Ncm. All implants were placed by the same experienced oral and maxillofacial surgeon under comparable clinical settings.

### 2.2. Surgical Procedures

#### 2.2.1. Treatment Protocol & Standardization

All patients were treated according to a standardized clinical protocol by the same surgeon (Prof. DT). This included:

- Preoperative CBCT-based planning
- Atraumatic extraction with socket curettage
- Grafting with Puros^®^ allograft and CopiOs^®^ membrane
- Delayed implant placement based on radiographic healing
- Digital prosthetic workflow using intraoral and facial scans
- Prosthetic restorations were completed by a single prosthodontist (PG) Patients received oral hygiene instructions and were enrolled in a recall program including clinical and radiographic follow-up at 3 and 6 months post-restoration.

#### 2.2.2. Materials Used (Table 1)

Allograft: Puros^®^ demineralized bone matrix, ZimVie Inc., Westminster, CO, USA

Membrane: CopiOs^®^ Pericardium Membrane, ZimVie Inc., Westminster, CO, USA

Sutures: Prolene^®^ 6-0, Johnson & Johnson, Raritan, NJ, USA

Anesthetic: Articaine 4% with 1:200,000 epinephrine, 3M ESPE, St. Paul, MN, USA

Implants: See full list in Section 2.1

Mouth rinse: Chlorhexidine 0.2%, GlaxoSmithKline, Münchenbuchsee, Switzerland

Antibiotics: Amoxicillin/Clavulanate 1 g BID for 5 days

Analgesia: Mefenamic acid 100 mg, Pfizer, Vienna, Austria.

**Table 1:** Materials used for socket preservation.

Material	Product Name	Manufacturer	Function/Role
Allograft	Puros^®^ Demineralized Bone Matrix (DBM)	ZimVie Inc., Westminster, CO, USA	Osteoconductive & osteoinductive scaffold
Membrane	CopiOs^®^ Pericardium Membrane	ZimVie Inc., Westminster, CO, USA	Barrier membrane for guided bone regeneration
Sutures	Prolene^®^ 6-0	Johnson & Johnson, NJ, USA	Wound closure
Anesthetic	Articaine 4% with 1:200,000 epinephrine	3M ESPE, St. Paul, MN, USA	Local anesthesia
Mouth rinse	Chlorhexidine 0.2%	GlaxoSmithKline, Münchenbuchsee, Switzerland	Antiseptic oral rinse
Antibiotic	Amoxicillin/Clavulanate 1 g BID, 5 days	Ratiopharm, Ulm, Germany	Postoperative infection prophylaxis
Analgesic	Mefenamic acid 100 mg	Pfizer, Austria	Postoperative pain management

#### 2.2.3. Tooth Extraction

**Figure 1:**
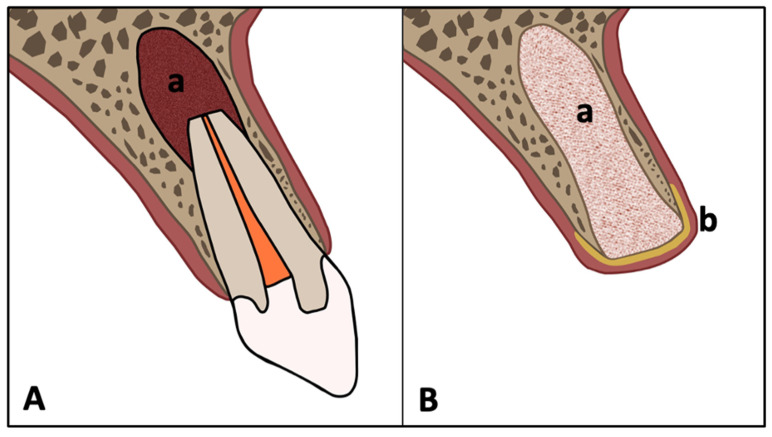
(**A**). Non-restorable tooth due to apical periodontitis or cyst (a = apical periodontitis). (**B**). Extraction sockets were aggressively curetted, filled with allogeneic bone substitute (Puros^®^) (a), and covered with pericardium membrane (CopiOs^®^) (b).

**Figure 2:**
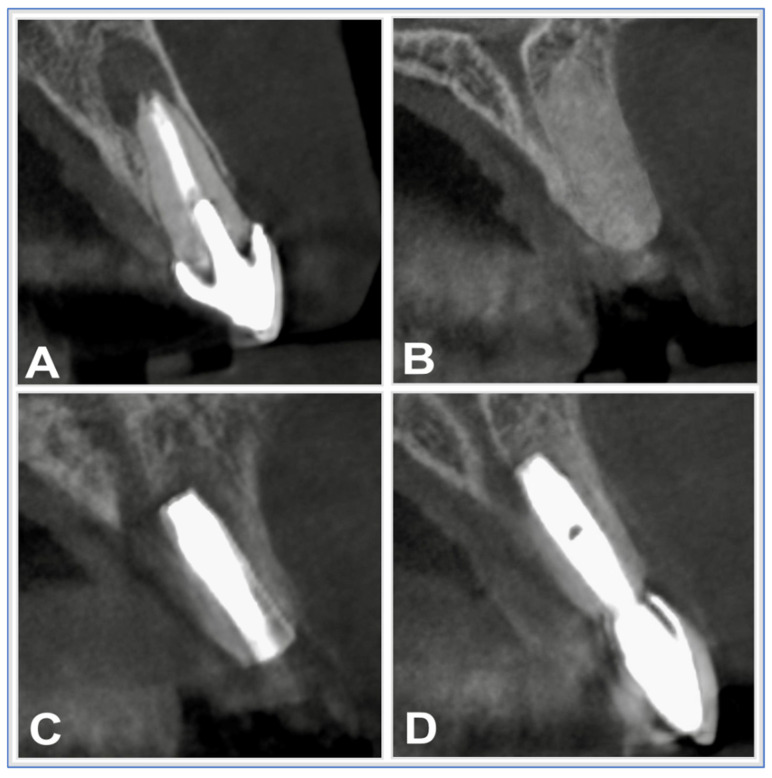
(**A**): Non-restorable tooth due to apical periodontitis or cyst. (**B**): Extraction sockets were aggressively curetted, filled with allogeneic bone substitute (Puros^®^), and covered with pericardium membrane (CopiOs^®^). (**C**): Placement of the dental implant. (**D**): Fully veneered zirconia crowns were fabricated for functional and aesthetic optimization.

Surgical extraction for apical periodontitis or cystic lesions required CBCT-based preoperative diagnostics and thorough medical history review. Local anesthesia was administered via infiltration or nerve block using articaine 4% with 1:200,000 adrenaline (3M ESPE, St. Paul, MN, USA). A full-thickness mucoperiosteal flap was raised, followed by osteotomy using rotary or piezosurgical instruments. In multi-rooted teeth, roots were sectioned to facilitate atraumatic removal. Residual root fragments were retrieved using periotomes or forceps (Figure 1A and Figure 2A).

#### 2.2.4. Socket Preservation

Extraction sockets were thoroughly debrided and grafted using a demineralized freeze-dried bone allograft (DFDBA; Puros^®^, ZimVie, Palm Beach Gardens, FL, USA), which consists of human donor bone processed via the Tutoplast^®^ method. This technique removes immunogenic components while preserving the native collagen matrix, supporting osteoconductive and osteoinductive properties. The graft material was covered with a resorbable pericardium membrane (CopiOs^®^, ZimVie), which serves as a barrier to prevent soft tissue ingrowth and maintain space for bone regeneration.

Socket preservation was performed using this standardized protocol in all patients. The decision to proceed with grafting was based on clinical indications such as buccal plate deficiency, periapical bone loss, or anticipated esthetic requirements. Implant timing was determined on a case-by-case basis depending on radiographic bone density, soft tissue condition, and patient-specific scheduling constraints.

Implant placement timing was guided by CBCT radiodensity assessment, clinical parameters, and surgeon experience. Patient scheduling constraints contributed to variability in time intervals.

#### 2.2.5. Implant Placement

Under local anesthesia, implants were placed using standard drilling protocols and minimal insertion torque (>30 Ncm). Mucoperiosteal flaps were sutured with 6-0 Prolene (Johnson & Johnson, NJ, USA) using interrupted technique. Postoperative care included chlorhexidine mouthwash (0.2%, Glaxo Smith-Kline, Geneva, Switzerland), antibiotic therapy (1 g amoxicillin/clavulanate BID for 5 days), and analgesia (100 mg mefenamic acid, Pfizer, Austria). Sutures were removed after 10 days, and no complications were reported during follow-up (Figure 2C). All implants were placed by the same experienced oral and maxillofacial surgeon under comparable clinical settings.

#### 2.2.6. Prosthetic Rehabilitation

Using a digital transfer system (Zirkonzahn, Gais, Italy), CBCT data, intraoral scans (S600 ARTI), and facial scans (Face Hunter) were combined in CAD/CAM software 2.0 for photorealistic modeling. Fully veneered zirconia crowns were fabricated via CAD/CAM (Reality Mode module, Zirkonzahn) to ensure both functional and esthetic restoration (Figure 2D). Patients received comprehensive oral hygiene instructions and were enrolled in a maintenance program for long-term follow-up.

### 2.3. Histopathological Processing and Evaluation

Periapical soft tissue was surgically collected at the time of tooth extraction under sterile conditions. The tissue was placed immediately into 4% buffered formalin and labeled with patient ID and date. All specimens were processed and analyzed in the in the ADK Diagnostics (Vienna, Austria). Decalcification and sectioning followed standardized protocols as described. Each case was scored by two independent pathologists using the DAIS system. Discrepancies were resolved by consensus.

Tissue samples were fixed in 4% buffered formalin for ≥24 h. Soft tissue was separated from any attached hard tissue, which was decalcified in Osteomoll^®^ (4% formaldehyde + 10% HCl) for ≥6 h at room temperature. Specimens were processed via automated embedding (e.g., VIP6AI^®^, ASP300S^®^, Histocom ExcelsiorAS^®^), paraffin-embedded, sectioned (2–3 μm), and stained with hematoxylin and eosin (HE). Barcode-tracked processing ensured high reproducibility. Polarized light was used to identify material deposits; additional stains were employed if bacterial colonization was suspected.

**Figure 3:**
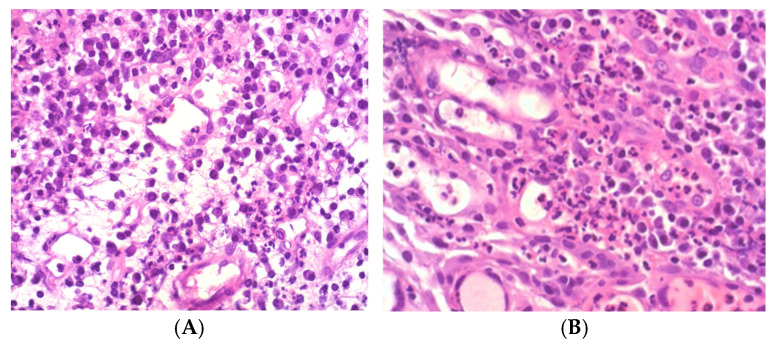
Apical lesions were scored DAIS 1–4 following final histological diagnosis. ((**A**): DAIS 2; (**B**): DAIS 4).

Histological diagnoses were scored using the DAIS system:

- DAIS 1: Low acute, low chronic inflammation
- DAIS 2: Low acute, high chronic
- DAIS 3: High acute, low chronic
- DAIS 4: High acute, high chronic (Figure 3)

The DAIS scoring was performed by two independent pathologists with experience in oral histopathology. Both examiners were blinded to clinical data. In cases of discrepancy, a consensus diagnosis was reached through joint review using light microscopy.

No formal sample size calculation was performed due to the retrospective nature and exploratory intent of this study. The total number of eligible patients within the study period (January 2020 to March 2024) was included to maximize data availability and strengthen subgroup analysis.

### 2.4. Data Processing

Data cleaning included removal of incomplete records, standardization of date formats, and recalculation of procedural intervals. DAIS categorization was verified against original histology reports. The DAIS score was assigned based on histopathological analysis of periapical soft tissue obtained during tooth extraction. As such, the evaluation of inflammatory severity preceded both implant placement and prosthetic rehabilitation in all cases.

Procedural timelines—specifically the extraction-to-implant and extraction-to-restoration intervals—were extracted from operative reports, digital treatment records, and the electronic patient management system. All dates were cross-verified by two independent reviewers to ensure consistency and accuracy.

### 2.5. Statistical Analysis

No formal a priori sample size calculation was performed due to the retrospective and exploratory design of the study. Instead, all eligible cases within the study period were included to maximize data availability. However, the markedly uneven distribution of DAIS categories—particularly the very low numbers in DAIS 1–3 compared with DAIS 4—represents an important limitation. This imbalance reduces the statistical power of subgroup comparisons and may affect the validity of ANOVA results. Therefore, all findings should be interpreted with caution.

Two separate one-way ANOVAs were conducted to compare procedural intervals (extraction-to-implantation and extraction-to-restoration) across DAIS groups 1, 2, and 4. Assumptions of normality (Shapiro–Wilk test) and homogeneity of variances (Levene’s test) were verified. Tukey’s HSD post hoc tests were used for pairwise group comparisons.

Chi-square tests assessed associations between DAIS scores and categorical variables (histology, jaw region, endodontic treatment, sex). Low-frequency histological categories were collapsed into an “Other” group to improve statistical validity.

Pearson’s correlation analysis was used to assess the relationship between age and DAIS score.

Although Shapiro–Wilk indicated deviations from normality for the DAIS 4 group (*p* = 0.002 for extraction-to-implantation; *p* = 0.005 for extraction-to-restoration), ANOVA was retained as the primary test because Levene’s tests supported homogeneity of variances (*p* = 0.791 and *p* = 0.894). One-way ANOVA is considered robust to moderate deviations from normality when variances are equal.

To account for potential bias due to non-normal distributions and uneven group sizes, sensitivity analyses were performed using Welch’s ANOVA and the non-parametric Kruskal–Wallis test.

## 3. Results

### 3.1. Demographic Characteristics

The study included 87 patients, with a mean age of 56.4 years (SD = 13.1) and a median age of 57 (Table 2). The majority of cases were classified as DAIS 4 (71.3%), while DAIS 3 was represented by only one patient (1.1%). The age distribution was centered between 50 and 60 years, characterizing a predominantly older patient cohort. A scatterplot revealed no clear association between patient age and the procedural intervals, suggesting that age had no substantial effect on the timing between tooth extraction and prosthetic rehabilitation.

The cohort included 39 male and 48 female patients. A total of 46 extractions were performed in the maxilla and 41 in the mandible. Tooth-specific extractions were as follows: Maxilla—11 (8), 12 (1), 13 (2), 14 (1), 15 (3), 16 (6), 17 (2), 21 (1), 22 (2), 23 (1), 24 (3), 25 (5), 26 (8), 27 (5) Mandible—31 (2), 32 (2), 34 (2), 35 (3), 36 (12), 37 (2), 42 (2), 43 (2), 44 (2), 45 (1), 46 (6), 47 (3) (Table 3).

Of the extracted teeth, 53 had undergone endodontic treatment and 34 had not. Histopathological diagnoses included 68 radicular cysts, 13 additional radicular cyst fragments, 4 periapical granulation tissues, and 1 follicular cyst (Table 4).

DAIS classifications were as follows:

- DAIS 1: 8 patients
- DAIS 2: 14 patients
- DAIS 3: 1 patient
- DAIS 4: 64 patients

Implant systems used in the study included:

- 3iT3 (n = 28)
- 3iT3PT (n = 17)
- Straumann BLX (n = 14)
- BEGO S (n = 12)
- SDS (n = 3)
- Biomet Boss 3i (n = 2)
- BEGO SCX (n = 1)
- BEGO SC (n = 1)
- Straumann BL (n = 1)

### 3.2. Extraction-to-Implantation Interval

A one-way ANOVA revealed no significant differences in the time from extraction to implant placement among DAIS groups (F(2, 83) = 0.338, *p* = 0.714). Mean time intervals were:

- DAIS 1: 174.38 days
- DAIS 2: 224.43 days
- DAIS 4: 207.67 days

Assumption checks:

- Normality: Shapiro–Wilk *p*-values: DAIS 1 = 0.331; DAIS 2 = 0.589; DAIS 4 = 0.002
- Homogeneity of variances: Levene’s test, *p* = 0.791

**Figure 4:**
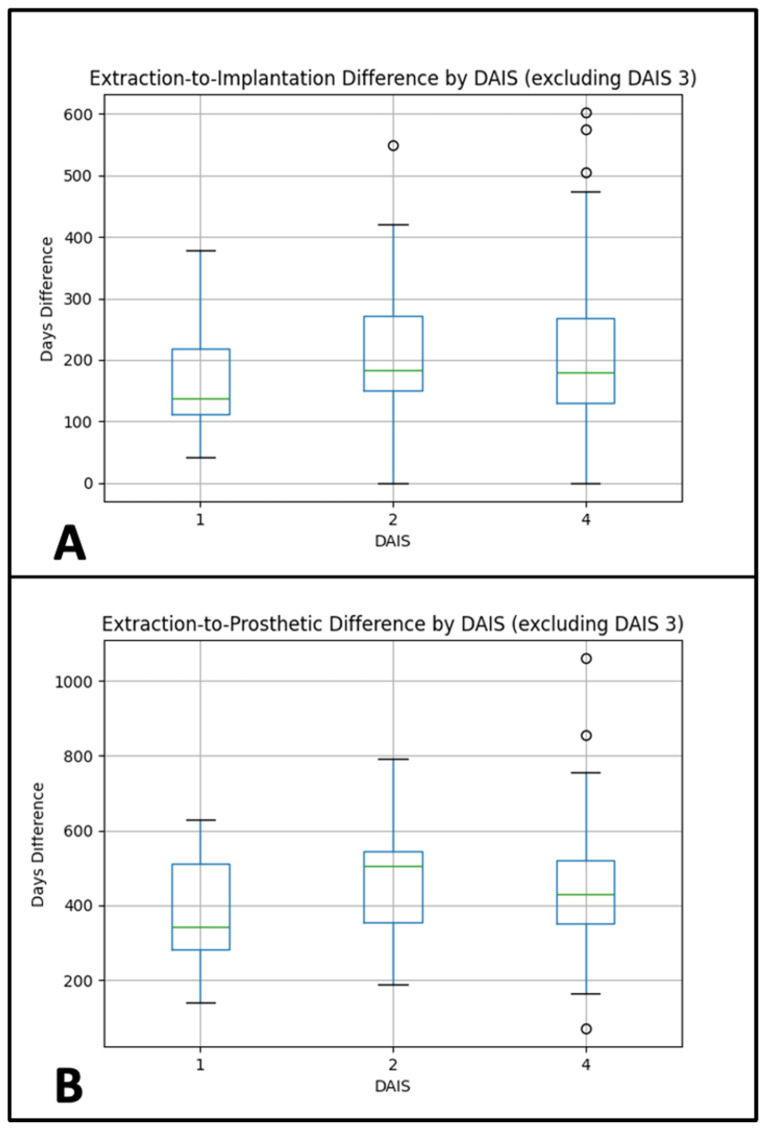
(**A**): Extraction-to-Implantation Time Differences. (**B**): Extraction-to-Prosthetic Time Differences.

Interpretation: The mean interval between extraction and implant placement did not differ significantly across DAIS groups. Post-hoc pairwise comparisons with Tukey’s HSD also showed no significant differences between any groups.
Sensitivity analyses. Additional testing with Welch’s ANOVA and the non-parametric Kruskal–Wallis test confirmed the ANOVA results, indicating no significant group differences in extraction-to-implantation time. See Figure 4A.

### 3.3. Extraction-to-Prosthetic Interval

A one-way ANOVA did not identify significant differences in the time from extraction to final prosthetic restoration across DAIS groups (F(2, 83) = 1.016, *p* = 0.367). Mean intervals were:

- DAIS 1: 375.38 days
- DAIS 2: 480.07 days
- DAIS 4: 452.03 days

Assumption checks:

- Normality: Shapiro–Wilk *p*-values: DAIS 1 = 0.382; DAIS 2 = 0.899; DAIS 4 = 0.005
- Homogeneity: Levene’s test, *p* = 0.894
  Post hoc: Tukey’s HSD test showed no significant pairwise differences.
  Interpretation: The mean interval between extraction and final prosthetic restoration also showed no significant variation across DAIS categories. Post-hoc comparisons again revealed no significant group differences.
  Sensitivity analyses. Welch’s ANOVA and Kruskal–Wallis testing supported the main findings, likewise indicating no significant differences across DAIS groups for extraction-to-prosthetic time. See Figure 4B.

### 3.4. Histological Diagnosis vs. DAIS Classification

A chi-square test revealed no significant association between histological diagnosis and DAIS classification (χ^2^(6) = 7.00, *p* = 0.321). Caution is advised due to low frequencies in some diagnostic categories.

## 4. Additional Analyses

### 4.1. Tooth Location (Maxilla vs. Mandible) vs. DAIS

**Figure 5:**
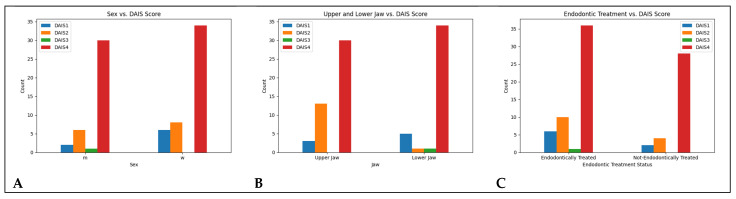
(**A**): Sex vs. DAIS. (**B**): Tooth localisation vs. DAIS. (**C**): Endodontic Treatment vs. DAIS.

A chi-square test showed a significant association between DAIS classification and jaw location (χ^2^(3) = 11.79, *p* = 0.008), indicating that localization may influence inflammation severity. DAIS 2 was more frequent in the maxilla, whereas DAIS 4 predominated in the mandible. See Figure 5B.

### 4.2. Endodontic Treatment vs. DAIS

No significant association was found between prior endodontic treatment and DAIS score (χ^2^(3) = 2.54, *p* = 0.468). See Figure 5C.

### 4.3. Age vs. DAIS

Pearson’s correlation analysis identified a weak but statistically significant positive correlation between patient age and DAIS score (r = 0.222, *p* = 0.039), suggesting a trend toward higher inflammation scores in older patients.

### 4.4. Sex vs. DAIS

No significant relationship was found between sex and DAIS score (χ^2^(3) = 2.63, *p* = 0.452). See Figure 5A.

## 5. Discussion

This study aimed to explore potential associations between procedural timing in implant-supported single-tooth restorations and the severity of apical inflammation as classified by the Dental Apical Inflammation Score (DAIS). The findings revealed no statistically significant differences in either the extraction-to-implantation or extraction-to-prosthetic intervals across DAIS groups, excluding the underrepresented DAIS 3 subgroup.

These results suggest that procedural timing, as implemented in routine clinical settings, may be largely independent of DAIS classification. Although some deviations from normality were observed—particularly in the DAIS 4 group—the homogeneity of variances remained intact, supporting the validity of the applied parametric tests. This implies that the observed lack of group-level differences was not due to statistical artifacts.

Importantly, no significant association was found between histological diagnosis and DAIS classification, even after aggregating low-frequency categories. This suggests that the histopathological profile of periapical lesions may not be a primary determinant of the inflammatory burden as quantified by DAIS, or that the study was underpowered to detect such a relationship.

A noteworthy observation was the significant association between tooth localization and DAIS classification. This finding may reflect anatomical differences between the maxilla and mandible, such as variations in trabecular density, vascularization, or healing dynamics. Additional studies focusing on the vascular architecture and perfusion patterns in both jaws may help clarify the biological mechanisms driving the observed association between jaw location and DAIS severity.

These structural factors could influence inflammatory resolution and tissue remodeling, thereby affecting DAIS outcomes.

Additionally, a weak but positive correlation between patient age and DAIS score was observed. This could be attributed to age-related changes in immune function, bone turnover, or chronic low-grade inflammation, all of which may affect periapical tissue responses. However, this association was modest and should be interpreted with caution.

Conversely, no significant associations were found between DAIS scores and either sex or prior endodontic treatment status. These findings suggest that gender-based physiological differences and prior root canal therapy do not meaningfully impact the severity of apical inflammation at the time of extraction.

The study has several limitations. The distribution of DAIS categories was highly unbalanced, with DAIS 3 particularly underrepresented, thereby reducing the strength of comparative analyses. Additionally, procedural intervals showed substantial overlap across DAIS groups, complicating efforts to define clinically meaningful thresholds for “low-risk” or “high-risk” timing. While lower DAIS scores were more frequently observed in cases with shorter procedural intervals (<30 days), the high variability in timing data limits the conclusiveness of this trend.

Although weak associations were noted between DAIS and both age and procedural timing, these correlations were not strong enough to support specific clinical recommendations. Nevertheless, the findings underscore the potential relevance of demographic and anatomical factors in periapical inflammation and call for further investigation into their roles.

### 5.1. Clinical Relevance and Impact on Implant Success

While previous literature has extensively discussed periapical pathology and the role of socket preservation in maintaining alveolar dimensions, little is known about how the histological severity of inflammation—captured by the DAIS—should influence the timing of implant placement. Our findings suggest that procedural timing may not differ substantially across DAIS categories, which challenges the assumption that higher inflammatory burden necessitates delayed intervention. This knowledge gap remains clinically important, as clinicians must often decide between early versus delayed implant protocols in sites affected by periapical pathology. Emphasizing DAIS within treatment planning could help clarify whether histological grading provides additional value for decision-making beyond anatomical and demographic factors.

Beyond the retrospective context of this study, the DAIS scoring system may have potential as a prognostic tool in clinical protocols. By providing a histopathological measure of inflammatory severity, DAIS could help stratify patients according to risk and guide treatment timing in implant dentistry. Future prospective studies are warranted to evaluate whether DAIS-based stratification can improve outcomes and be integrated into standardized decision-making algorithms.

### 5.2. Limitations

This study has several limitations. First, its retrospective design and reliance on existing records introduce potential selection bias and limit control over confounding factors. Second, the distribution of DAIS categories was markedly uneven, with DAIS 1–3 being underrepresented. This reduced the statistical power of subgroup comparisons and complicates generalizability. Third, deviations from normality in the DAIS 4 group raise caution regarding parametric inference. However, sensitivity analyses (Welch’s ANOVA and Kruskal–Wallis) confirmed the non-significant results, supporting the robustness of the main findings. Fourth, multiple implant systems from different manufacturers were used. Although all procedures followed a standardized protocol by the same surgeon, differences in implant geometry and surface properties may have influenced healing dynamics. Finally, the lack of long-term follow-up data on implant survival and peri-implant health, as well as incomplete documentation of systemic factors such as smoking or diabetes, limits broader extrapolation.

## 6. Conclusions

This retrospective study did not reveal a statistically significant association between the timing of implant-related procedures—specifically, the intervals from tooth extraction to implant placement and to final restoration—and the severity of periapical inflammation, as measured by the Dental Apical Inflammation Score (DAIS). While procedural timing did not significantly differ between DAIS groups, higher DAIS scores were weakly correlated with older age and were more frequently observed in mandibular sites. These findings suggest that anatomical and demographic factors may play a more prominent role in influencing inflammatory severity than procedural timing alone.

Further prospective studies are needed to clarify whether the timing of implant placement and prosthetic restoration influences healing dynamics, osseointegration, or long-term implant success in cases involving apical pathology.

## Figures and Tables

**Table 2:** Sex vs. DAIS.

Sex	DAIS 1	DAIS 2	DAIS 3	DAIS 4
m	2	6	1	30
w	6	8	0	34

**Table 3:** OK/UK vs. DAIS.

OK_UK	DAIS 1	DAIS 2	DAIS 3	DAIS 4
OK	3	13	0	30
UK	5	1	1	34

**Table 4:** Endodontic treatment vs. DAIS.

Endo_Treated	DAIS 1	DAIS 2	DAIS 3	DAIS 4
yes	6	10	1	36
no	2	4	0	28

## Data Availability

The original contributions presented in the study are included in the article, further inquiries can be directed to the corresponding author.

## Abbreviations

DAIS—Dental Apical Inflammation Score; CBCT—Cone Beam Computed Tomography; DVT—Digital Volume Tomography; STROCSS—Strengthening the Reporting of Cohort Studies in Surgery; DPU—Danube Private University; EMR—Electronic Medical Record; SPSS—Statistical Package for the Social Sciences; SD—Standard Deviation; df—Degrees of Freedom; ANOVA—Analysis of Variance; IRB—Institutional Review Board; STARD—Standards for Reporting of Diagnostic Accuracy; CI—Confidence Interval; OS—Oral Surgery.
